# Hybrid total-body pet scanners—current status and future perspectives

**DOI:** 10.1007/s00259-021-05536-4

**Published:** 2021-10-14

**Authors:** Vanessa Nadig, Ken Herrmann, Felix M. Mottaghy, Volkmar Schulz

**Affiliations:** 1 Department of Physics of Molecular Imaging Systems, Institute for Experimental Molecular Imaging, RWTH Aachen University, Aachen, Germany; 2 Department of Nuclear Medicine, University of Duisburg-Essen, Duisburg, Germany; 3 German Cancer Consortium (DKTK) - University Hospital Essen, University of Duisburg-Essen, Essen, Germany; 4 Department of Nuclear Medicine, University Hospital Aachen, RWTH Aachen University, Aachen, Germany; 5 Department of Radiology and Nuclear Medicine, Maastricht University Medical Center (MUMC+), Maastricht, The Netherlands; 6 Hyperion Hybrid Imaging Systems GmbH, Aachen, Germany; 7 Physics Institute III B, RWTH Aachen University, Aachen, Germany; 8 Fraunhofer Institute for Digital Medicine MEVIS, Aachen, Germany

**Keywords:** PET, CT, MRI, Total-body, Sensitivity, Long axial FOV

## Abstract

**Purpose** Since the 1990s, PET has been successfully combined with MR or CT systems. In the past years, especially PET systems have seen a trend towards an enlarged axial field of view (FOV), up to a factor of ten. **Methods** Conducting a thorough literature research, we summarize the status quo of contemporary total-body (TB) PET/CT scanners and give an outlook on possible future developments. **Results** Currently, three human TB PET/CT systems have been developed: The PennPET Explorer, the uExplorer, and the Biograph Vision Quadra realize aFOVs between 1 and 2 m and show a tremendous increase in system sensitivity related to their longer gantries. **Conclusion** The increased system sensitivity paves the way for short-term, low-dose, and dynamic TB imaging as well as new examination methods in almost all areas of imaging.

## Introduction

Tomographic imaging techniques for whole-body imaging such as magnetic resonance imaging (MRI), computed tomography (CT), and positron emission tomography (PET) have been enabled in the 1970s due to the invention of computer technology, which allowed image reconstruction within the clinic [19]. The size of the axial field of view (aFOV) of these early systems was often driven by the clinical need to capture one organ and the total cost of the system as well as hardware and computational complexity. As in many technology-driven areas, several developments focused on exploring novel applications and improving the performance of imaging devices, resulting in an improvement of the signal-to-noise ratio (SNR) and a faster acquisition in addition to artifact-free and high-resolution images. During this time, many groundbreaking inventions shaped the current state of imaging equipment. For instance, in the area of MRI, high-field MRI and parallel imaging with more than one radiofrequency (RF) coil were invented. CT quickly transitioned to higher spatial resolution with new multi-slice detectors and novel diagnostic methods such as spectral CT. The invention of time-of-flight (TOF) PET, first appearing in the 1980s [68, 104] and commercially introduced in 2006 by Philips [107], and the advent of the silicon photomultiplier (SiPM) [77] in combination with a modern readout architectures, which offer nearly 100 times more channels, were important milestones in the digital evolution of modern PET systems. Parallel to the hardware improvements for all modalities, the reconstruction methods rapidly developed towards iterative imaging reconstruction, which allowed a more precise modeling of the system properties. Recent developments in image analysis employ machine learning (ML) and deep learning (DL) methods for the utilization of prior information, including a high degree of automation [28, 111, 120]. In PET, the transition from photomultiplier tubes (PMTs) to SiPMs has enabled significant improvements in spatial and TOF resolution over the last decade. This process has been accompanied by an almost complete digitization of the PET electronics, resulting in increased system costs and complexity. In addition, the readout electronics of modern PET systems were developed to be scalable [84, 118]. Thus, recent technological developments of PET detectors seemed to be approaching a point where system geometries that differ from the conventional gantry approach are feasible [70, 98]. It took almost 20 years for these ideas to be taken up, finally leading to the first human total body (TB) PET systems, one of which is in the prototype stage [52, 109] and two others are even commercially available systems [25, 106]. In this paper, we focus on the evolution of PET systems and the underlying technology towards total-body (TB) PET systems. We will show the difference of these novel, highly sensitive systems and discuss their unique properties. Furthermore, we will discuss interesting methods and applications of current state-of-the-art TB PET systems as well as future developments that might have the potential to be transferred into clinical practice.

## Evolution of PET/CT and PET/MR systems

PET/CT or PET/MR systems employ dedicated detector blocks, most commonly arranged as a detector ring around the region of interest (ROI), consisting of three key components: A dense and fast-decaying scintillator to stop incident *γ*-photons and convert them into optical photons, a highly sensitive photo-sensor to detect the optical photon shower and generate a signal in form of a voltage or current pulse, as well as precise custom-designed readout electronics to trigger on these generated signals and digitize their timestamp and energy. These components are to be duplicated or re-scaled if realizing a hybrid TB PET system.

### Scintillators

Over the past decades, efforts have been made to exploit the properties of different kinds of scintillator materials, attempting to find an existing or even develop a new material that comprises a high light yield, a weak non-linearity of the *γ*-energy related to the generated number of optical photons, a high stopping power, and a fast scintillation decay time. These characteristics reduce the limitation of the TOF capabilities of a PET system by the scintillator [55, 73] and therefore contribute to the SNR gain of a (TB) PET system. Among scintillator materials such as sodium iodide (NaI:Tl), gadolinium oxyorthosilicate (GSO:Ce), and many others, bismuth germanate (BGO, Bi_4_Ge_3_*O*_12_) was treated as the most promising one due to its detection efficiency [63]. It quickly got replaced by lutetium-based and cerium-doped candidates, such as lutetium oxyorthosilicate (LSO, Lu_2_SiO_5_:Ce) and lutetium yttrium oxyorthosilicate (LYSO, (Lu-Y)_2_SiO_5_:Ce), about 20 years ago [54, 63, 81]. So far, no other material has been able to compete with the considerably higher light yield and detection efficiency of LYSO (up to 32,000 ph/MeV and a density of 7.2 g/cm^3^ [55]), while maintaining a comparably fast scintillation decay [81, 108, 113]. However, the use of BGO is currently reinforced, since it still comes along with a high detection efficiency and allows for the application of detection techniques making use of Cherenkov emission in TOF-PET systems [41]. In addition, the decreased cost of BGO could play an important role in selecting a suitable scintillator material for a TB PET system.

Apart from the material used, the employed scintillator geometry impacts the TOF resolution of a PET system. Long scintillator needles deteriorate the TOF resolution by light jitter, but are required in clinical PET systems to increase the system sensitivity. High-resolution (HR) scintillator arrays increase the spatial resolution of a PET system, while multi-layered and (semi-)monolithic ones allow for depth of interaction (DOI) positioning [17, 64, 66, 67, 92]. These are for sure advantages that will also transfer to TB PET architecture. Disadvantages could lie in growing crystal ingots to manufacture a large number of uniform scintillator blocks. However, production costs are likely to be reduced due to less effort when cutting the crystal blocks and the lack of a complicated assembly of small crystal needles to a matrix.

While scintillator material and geometry do not play a role in constructing hybrid PET/MRI systems, they are important components to consider for the integration of PET and SPECT or CT into one system. These systems require scintillation crystals that are highly absorbent for *γ*-photons in the energy range of 20 to 150 keV [55]. The scintillator material, e.g., the lutetium component of LYSO, being intrinsically radioactive will lead to distortions during image acquisition. With an increased amount of LYSO needed for a TB PET system, the background radiation distorting the image will increase. Nonetheless, acquiring PET and CT *γ*-photons with the same detectors is favored for highly integrated systems, e.g., as realized by two-layered phoswich detector blocks [94], reducing the need of additional space and acquisition time required for two separate imaging units and allowing for simultaneous image acquisition, motion correction, dose monitoring, and guided radiotherapy [63].

### Photo-sensors

**Fig. 1:**
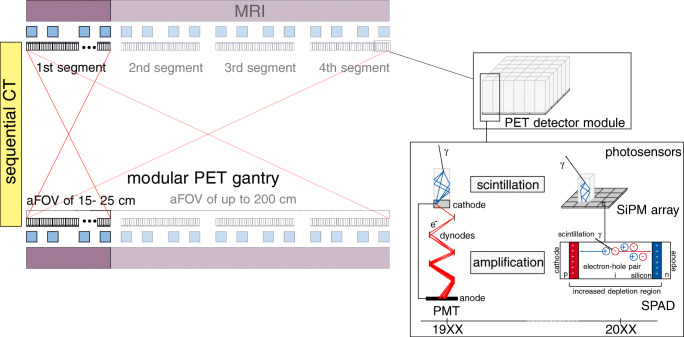
The evolution of PET system components, involving a transition to novel scintillator materials and new photo-sensor techniques, ultimately resulting in the usage of LYSO and SiPMs as one small detector unit in state-of-the-art PET systems. The readout electronics transitioned from mostly hardware-controlled solutions to highly integrated SPUs. Illustration of the photo-sensor techniques follows [81]

In the 1980s, early PET systems were built using PMTs in order to generate a signal in the form of a current pulse from incident optical scintillation photons by exploiting the photo-electric effect and amplifying the released electron to an electron shower using a cascade of dynodes (see Fig. 1). While this detection of low levels of light results in a sound SNR, PMTs suffer from a poor quantum efficiency (QE) of only 15 to 25%, in rare cases up to 40%, a low form factor, as well as a high sensitivity to magnetic fields [13, 65, 119].

For these reasons, they were soon competing with solid-state detectors, in particular photo-diodes. Firstly developed in the 1940s, photo-diodes are pn-junctions with a depletion region that are reversely biased to prevent a current flow unless an electron-hole pair is created by an incident optical photon [13]. With a QE of 60 to 80%, these diodes were much more suitable candidates to be employed in PET systems, but came along with a low SNR [81]. Increasing the depletion region by a high-field region, i.e., introducing the positive-intrinsic-negative (PIN) diode model, improved the sensitivity of the photo-diodes, but did not increase the diode current. The development of avalanche photo-diodes (APDs) in the 1950s marked a new generation of diodes, where the movement of the first electron-hole pair through the depletion region creates secondary electron-hole pairs, and thus amplifies the signal, still keeping it proportional to the number of detected optical photons [13] (see Fig. 1). The amplification also caused an increased sensitivity to voltage and temperature fluctuations. Following this evolution, single-photon avalanche diodes (SPADs) were developed, which are APDs operated in Geiger mode, i.e., reversely biased above their breakdown voltage, so that the hit of a single optical photon is sufficient to create a self-sustaining avalanche that has to be electrically quenched to make the diode sensitive again. SPADs assembled in arrays of several thousand pieces, known as silicon photomultiplier (SiPM) and firstly introduced in 1989, are commonly used in nowadays PET systems [13].

SiPMs quickly gained popularity in PET due to their compact design and their fast response to incident light, making them especially useful to preserve TOF information [12, 49, 108]. Coming along in different packaging sizes and form factors, they allow an easy and flexible re-scaling of a given detector geometry to larger, i.e., TB PET, system scales. Besides analog versions by different vendors, e.g., KETEK, SensL, HPK, Broadcom, or FBK, that are widely used in analog PET systems, Philips has developed a truly digital SiPM (dSiPM) about 10 years ago, which is used in Philips digital PET scanners [42, 93]. While dSiPMs outperformed their analog predecessors at first, analog SiPMs have seen tremendous improvements regarding their photon detection efficiency (PDE) in recent years, reaching PDEs of up to 55% [41]. In existing TB PET systems, both the analog and digital technologies were able to set foot. As a big advantage to PMTs, APDs and SiPMs are insensitive to magnetic fields and therefore enormously facilitated the integration of PET and MRI into one hybrid system, allowing for simultaneous imaging of anatomical and metabolic information without the additional radiation dose of a SPECT or CT acquisition [12, 21, 80, 108].

### Detector data acquisition and processing

In the early days of PET, data acquisition and processing were done in an analog and hardware-controlled manner. This ranged from event positioning on the detector, combining trigger groups of photomultipliers, energy measurements up to the coincidence pairing of events. However, this has changed substantially during the last decades, as the entire event processing chain has become digital and software-driven. Local digitization at detector level was a key factor for improving the energy resolution and especially the timing resolution of modern PET systems. Besides the strong need for local digitization, the development of new readout electronics was also triggered by the high granularity of solid-state detectors such as SiPMs (see section “Photo-sensors”), which require the readout and processing of nearly 100 times more individual readout channels [74].

**Fig. 2:**
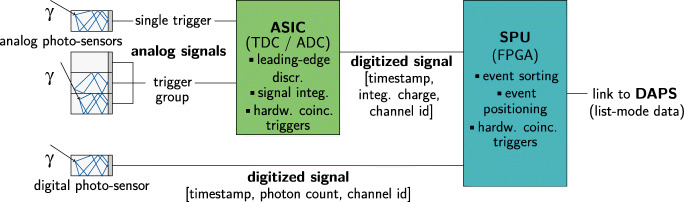
Schematic drawing of the readout architecture in modern PET systems. Analog signals from single or groups of photo-sensors are digitized by an ASIC employing analog- and time-to-digital converters (ADCs/TDCs) and then routed to an SPU. A digital SiPM already digitizes the trigger itself. The SPU commonly houses an FPGA for first event sorting and processing steps before the digitized event information is processed on a data acquisition and processing server (DAPS). Here, detected single events or already matched coincidences are given in list-mode format

Directly after the analog detector block, application-specific integrated circuits (ASICs) are employed to trigger on the analog signal pulses created by SiPMs and PMTs (see Fig. 2) [18, 72, 91, 96]. Alternatively, digital SiPMs are used which are combining the function of an analog SiPM and the ASIC [16, 95]. In both cases, the transferred information needs to be collected and processed by so-called singles processing units (SPUs) [117]. Central elements of these boards are field-programmable gate arrays (FPGAs), which allow for a parallel processing of the events registered by individual detector channels. While physical signals between detector blocks and SPUs could be analog [90, 97] or digital [16, 117], depending on the technology involved, the output exported from the SPUs is typically digital, often using Ethernet connectors and protocols. Hence, nowadays, detectors have became autonomous digital data collection units, acting as local nodes in a larger detector network. Tree-like network topologies are often used, which allow the connection of individual detector elements or blocks via dedicated data collection boards.

In order to form coincidence events out of the digital stream of singles, several vendors are using so-called hardware coincidence units, which combine two coincident singles on hardware level. It has been published that modern readout architectures have implemented this hardware coincidence unit entirely in software due to the constantly increasing performance of computers [36, 37, 62]. This allows a higher degree of freedom in processing the data, in particular to use varying acceptance angles or more advanced methods from statistics or machine learning [20, 29, 67].

With the introduction of a scalable readout architecture, which is offered by various vendors and research groups, system geometries other than the conventional ones have become possible, such as dedicated breast PET or PET/MR systems and whole-body (WB) PET/CT systems [79, 98, 110]. The detector and readout technology was thus ready to be scaled up to TB PET systems. From a mechanical point of view, this seems to be a trivial duplication of detector rings. However, the electronic readout infrastructure of a TB PET system faces a number of new challenges that are not present in WB PET systems. These are axially limited coincidence windows, a way of managing the overall increased data rate; related to this, the need for a higher computing power for the coincidence search as well as a larger and more complex cooling and power supply infrastructure, just to name a few.

## Hybrid total-body PET systems—status quo

Efforts to integrate PET and morphological imaging methods, like CT or MRI, have been undertaken since the late 1990s [69, 112]. In the past two decades, not only technological challenges such as coping the interference of MR and PET electronics or reducing the CT radiation dose, but also infrastructural demands regarding the available space, the required power supply, and available financial means for clinical installations have been in constant interplay with the necessity to enlarge the bore diameter for patient comfort as well as the need to elongate the aFOV to increase the PET system’s sensitivity and reduce the scan duration.

### From step-and-shoot to single-shot acquisition

Limited by the aFOV width of standard PET/CT and PET/MR scanners, a translation of the patient bed with slightly overlapping aFOV positions along the axial path is necessary to cover the patient’s whole torso (imaging “from eyes to thighs”) or even whole body during image acquisition. Reconstructing an image from the acquired slices may result in quantification errors due to varying noise levels in regions with different uptake [51]. With PET systems achieving lower CRTs and being able to acquire TOF information, the standard process of moving the patient bed to discrete positions, called step-and-shoot (SS) acquisition, could be reduced to shorter time frames per bed position and, finally, is about to be replaced by a so-called single-shot or continuous-bed-mode (CBM) acquisition [51].

**Fig. 3:**
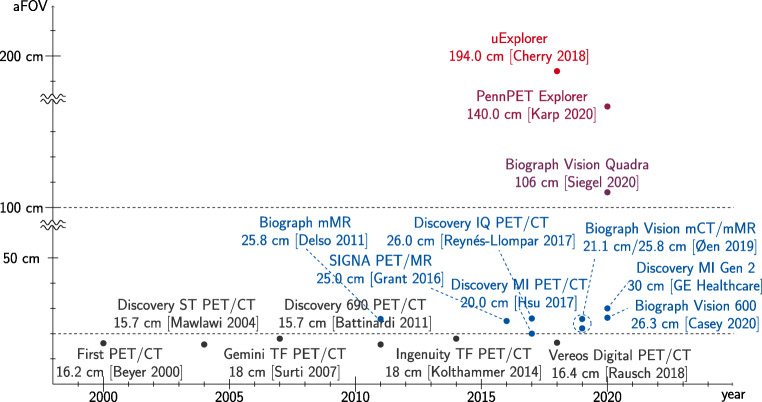
Evolution of the aFOV of commercial and research multi-modal human PET systems. The timeline uses the dates of the first available reports, performance studies and NEMA (National Electrical Manufacturers Association) characterizations of the respective system. A slight delay to the actual product launch is therefore possible. The presented information has been extracted from [6, 11, 22, 25, 30, 35, 38, 46, 52, 53, 60, 76, 86, 88, 100, 107]

Overall, the aFOV width has seen a trend towards larger axial coverage (see Fig. 3), resulting in fewer bed positions that need to be imaged. Standard multi-modal PET system reached aFOVs more than 20 cm in the second half of the last decade, adding about 50% to the aFOVs of the early years of this century. Next to the described enhanced detection efficiency of the emerging SiPM detector technology, this evolution contributed to a remarkable shortening of the acquisition time due to acquiring more coincidence events in the same time frame. The current PET/CT systems can acquire a whole-body image within less than 10 min, obviously dependent on the injected dose. Within the late 2010s, a new generation of PET scanners has entered the field, exploiting the benefits of recent developments in electronic and material research to realize aFOVs of 1 to 2 m. These scanners allow whole-body acquisition within less than 60 s and thereby will give a real time insight into (patho-)physiological processes. This potential is further discussed in Section “Potential clinical applications”.

### The challenge of scaling hybrid whole-body systems

Scaling a WB PET system to the length of a TB system requires the system electronics to be able to process a significantly higher data rate stemming from the increased number of PET modules. Additionally, reconstruction algorithms have to be adapted to reduce artifacts from increased parallax errors due to the longer aFOV [52]. Scaling a hybrid WB system holds further challenges. While PET/CT scans are still acquired sequentially exploiting the short CT scan duration in the order of few seconds, PET/MRI scans have to be conducted truly simultaneous to keep the total scan duration to a minimum (both a PET and MRI scan can last up to 30 min each) and benefit from gated correction methods [114].

Current commercial PET/CT scanners of any aFOV length employ CTs positioned in-line to the PET bore (e.g., [34, 82, 102], see Fig. 1), allowing the patient table to be moved through both gantries, avoiding a relocation of the patient and thus preventing movement artifacts. Integrating PET and MR electronics into the same bore requires sophisticated shielding of the PET electronics as well as a highly integrated design of the electronic and infrastructural components such as cooling or power supply. State-of-the-art PET inserts and PET/MRI systems therefore employ dedicated housings with carefully chosen shielding for PET electronics and exploit design ideas such as RF-penetrable PET electronics, integrating RF screens into the PET detectors and positioning the PET electronics in a split-gradient coil [4, 39, 40, 83, 117, 121].

For PET/CT systems, enlarging the aFOV of the PET system from a mechanical point of view can be achieved by adding detector rings to the PET gantry (see Fig. 1). The increased costs of adding detector rings to the PET system are tried to overcome by design approaches reducing the number of PET detector elements and positions to the required minimum, e.g., in a checkerboard pattern or other sparsely covered cylindrical geometries by omitting single rings or slices [7, 114]. This approach will clearly lead to a reduced sensitivity of the PET system. However, in a TB PET system, the increased axial coverage may be able to overcome this effect and keep the sensitivity at a level sufficient for fast and low-dose scans.

While TB PET/CT systems have already been made commercially available [25, 106], TB PET/MRI systems are yet to be designed. Big advantages of replacing the CT component of a TB system by an MRI are the better soft tissue contrast, a lower radiation exposure, and the possibility of continuous MR- or PET-based motion compensation coming along with the use of MRI [116].

### Research prototype total-body PET/CT systems

Currently existing TB PET research systems are the PennPET Explorer (UPenn, Philadelphia, Pennsylvania, USA; KAGE Medical, Wayne, Pennsylvania, USA; Philips Healthcare, Highland Heights, Ohio, USA) intended to be used for human patients, and two smaller systems, the miniExplorer scanners (UC Davis, California, USA, and UC Davis School of Veterinary Medicine, California, USA) designed for pre-clinical imaging of non-human primates. Additionally, miniExplorer II allows for the imaging of the human brain and has been used in studies with canine patients [58]. All these scanners were developed by the EXPLORER consortium (EXtreme Performance LOng REsearch ScanneR [58]). Both miniExplorer scanners realize an aFOV of about 45 to 48 cm and are based on the components of larger human PET systems. MiniExplorer I makes use of the components of the Siemens mCT (Siemens Medical Solutions, Knoxville, Tennessee, USA), while miniExplorer II is based on the components of the uExplorer [8, 58]. Using a variable acceptance angle, studies with miniExplorer I have shown the immense gain in system sensitivity coming along with the coverage of a larger solid angle [8].

The PennPET Explorer focuses on increased TOF performance, reaching CRTs of down to 250 ps, and functional scalability of the aFOV with respect to the application (currently 64 cm, up to 140 cm are planned) [25, 52]. Further specifications of the currently existing research systems can be found in Table 1.

**Table 1:** Overview on existing TB PET/CT commercial and research systems

System	Biograph Vision Quadra	uExplorer	PennPET Explorer	miniExplorer I	miniExplorer II
Company/facility	Siemens Healthineers	UC Davis and United Imaging Healthcare	UPenn, KAGE Medical and Philips	UC Davis	UC Davis School of Veterinary Medicine
Purpose	Clinical (human)	Clinical (human)	Clinical (human)	Pre-clinical (non-human primates)	Pre-clinical
Bore diameter (cm)	78	76	n.a.	43.5	52
aFOV (cm)	106	194	64 (140 planned)	45.7	48.3
tFOV (cm)	n.a.	68.6	57.6	32	n.a.
Photo-sensors	Analog SiPMs	Analog SiPMs (SensL)	Digital SiPMs (PDPC)	PMTs	Analog SiPMs (SensL)
Scintillators	LSO (20 mm)	LYSO (18.1 mm)	LYSO (19 mm)	LYSO (20 mm)	LYSO (18.1 mm)
CTR (ps)	219	430	250	609	409
dE/E (%)	10.1	11.7	12.0	n.a.	11.7
Spatial res. (mm)	3.4	2.9	4.0	3.0	2.61
Sensitivity (kcps/MBq)	174	174	55	n.a.	51.8
NECR / Mcps (kBq/cc)	< 2.5 (26)	< 1.855 (9.6)	> 0.001 (30)	n.a.	>

The graphic reports system characteristics and performance parameters such as bore and gantry measures, detector components, energy resolution, timing resolution, spatial resolution, sensitivity, and noise equivalent count rate. Performance parameters and system characteristics have been taken from [5, 8, 25, 52, 58, 78, 87, 100, 102, 105, 123]

### Total-body PET/CT systems in clinical routine

Commercial TB PET systems were first made available as combined TB PET/CT systems in the past 2 years. They involve a human TB PET/CT system, the uExplorer (UC Davis, California, USA; United Imaging Healthcare, Shanghai, China) that is already used in clinical research and intended to be used in clinical practice (see Table 1). While this scanner was also developed by the EXPLORER consortium, it shows significant differences in its layout compared to the research systems. The uExplorer realizes a longer aFOV of almost 2 meters and therefore benefits from a 40-fold effective sensitivity gain imaging the whole body of a patient (24-fold imaging the corpus, 4- to 5-fold imaging specific organs) that results in an immense reduction of the acquisition time and administered tracer dose [25]. Alternatively, the SNR of a PET image could be improved, keeping the scan time and dose as in a standard PET scan. The SNR changes related to the square root of the effective sensitivity, meaning a 40-fold sensitivity gain is equal to an approximately 6-fold SNR gain [25]. For organ-dedicated PET scans, this results in the rather low increase of the SNR by a factor of about 2, assuming a 4- or 5-fold effective sensitivity gain. It has to be investigated whether this justifies the increased costs and additional spatial requirements of the uExplorer.

**Fig. 4:**
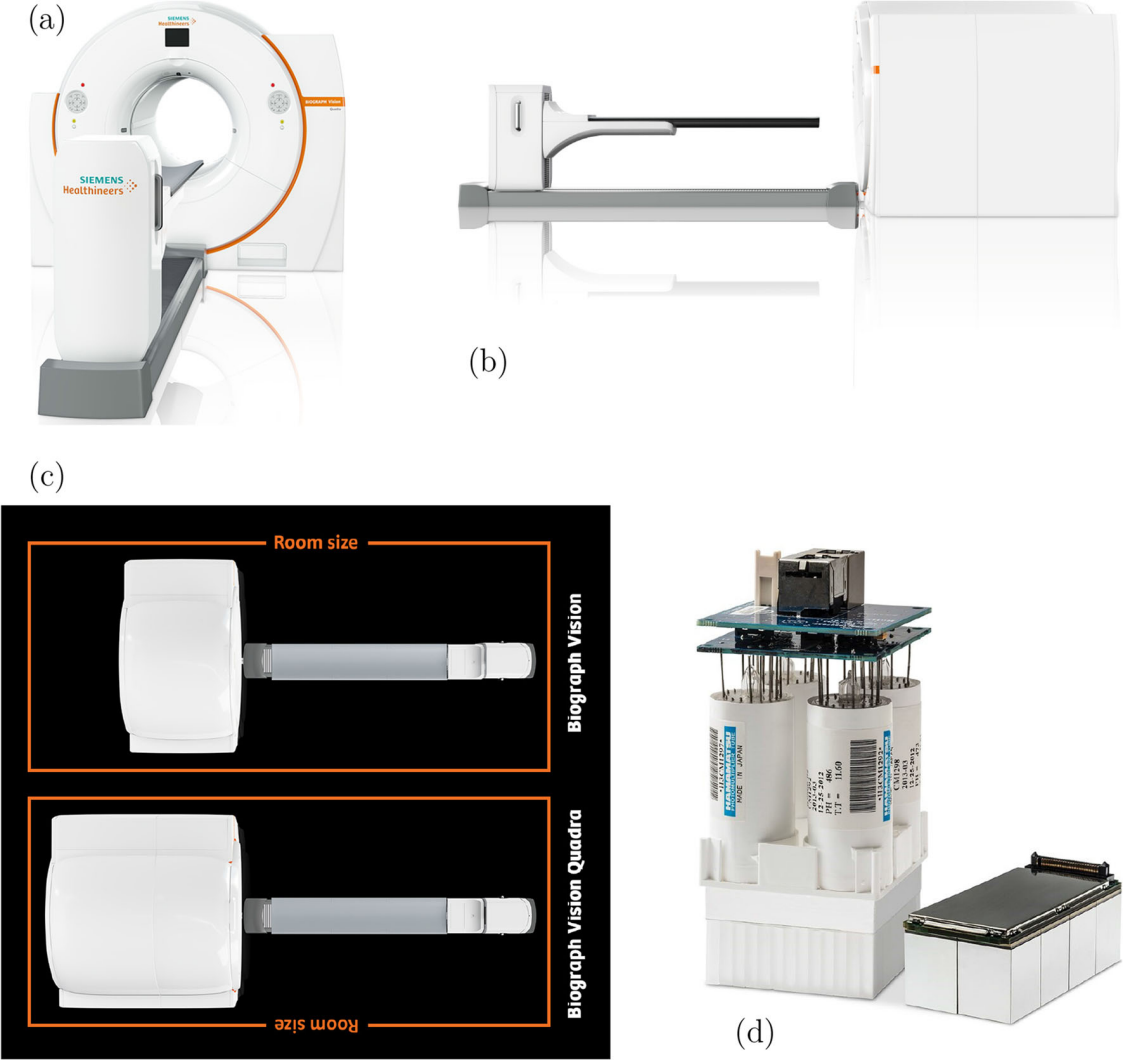
Siemens Biograph Vision Quadra. **a** Front view. **b** Side view. **c** Top view and comparison of the footprint with the predecessor Biograph Vision. **d** Optiso UDR detectors. Pictures have been taken from [103] with kind permission of Siemens Healthineers

**Fig. 5:**
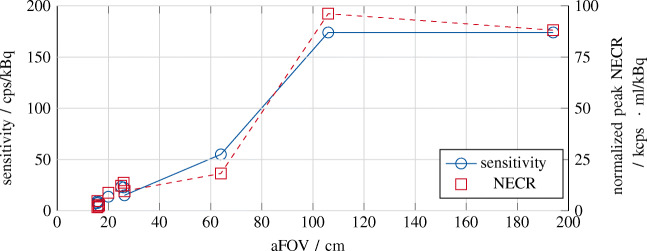
Sensitivity and NECR of existing PET/CT systems (see Fig. 3) depending on their aFOV. The NECR was normalized according to the activity concentration used in the FOV. Data have been taken from [6, 11, 38, 46, 52, 60, 86–88, 100, 105]. Lines have been added to the data points to guide the eye

Very recently, as a first competitor to the uExplorer, Siemens Healthineers (Siemens Medical Solution USA, Knoxville, Tennessee, USA) announced their first commercial TB PET system [106]. This prototype with an aFOV of 106 cm is a ring-based extension of the Siemens Biograph Vision 600 and intended as a PET/CT tomograph (see Fig. 4) [100, 103]. It exceeds the TOF performance of most of the Explorer scanners by about 200 ps and is able to compete with the PennPET Explorer scanner by showing an about 30 ps lower CRT (see Table 1). Even with only a fourfold extension of the aFOV, so in total about half the aFOV of the uExplorer scanner, the tomograph is expected to accomplish significant improvements regarding scan duration and dose reduction, e.g., in pediatrics, while maintaining the required footprint size to fit into current clinical imaging facilities (see Fig. 4c), which makes it a more suitable candidate to be included into clinical routine than the uExplorer. A first clinical study assessing these benefits has been performed based on an intra-individual comparison of patients undergoing examination with both the Biograph Vision 600 and the Biograph Vision Quadra [1]. Compared to its predecessor at Siemens, the sensitivity in the center of the FOV of the Biograph Vision Quadra was increased by a factor of about 11.5 from 15.1 to 174 kcps/MBq. As expected, the peak noise equivalent count rate (NECR) increased by about almost the same factor from 296 kcps measured at 30.9 kBq to 2.5 Mcps measured at 26 kBq [87, 100]. Figure 5 compares the sensitivity and NECR of existing PET/CT systems depending on their aFOV. Compared to the Biograph Vision Quadra, the other commercial TB PET system, i.e., the uExplorer, achieves about the same sensitivity of 174 kcps/MBq despite an aFOV twice as large [105]. This indicates that the sensitivity gain related to the aFOV saturates for aFOVs reaching a certain length as it was shown for point and line sources [27, 101]. However, the scanning procedure according to NEMA standards may contribute to this impression. A 70-cm line source does not completely fill the aFOV of the uExplorer, but of the Biograph Vision Quadra. It is expected that the uExplorer would outperform the Biograph Vision Quadra regarding its sensitivity in case of a longer line source being used. Still, a lower sensitivity of 147 kcps/MBq is reported for a line source of 170-cm length [105]. In contrast, the effective sensitivity may benefit from the TOF performance of the Biograph Vision Quadra (CRT of 219 ps compared to a CRT of 430 ps for the uExplorer, see Table 1), leading to a higher SNR.

### Potential clinical applications

The superior sensitivity of TB systems can be clinically exploited in various different ways. The faster image acquisition facilitates a higher throughput. Additionally, application of less radioactivity allows for PET studies to be performed more frequently during the course of a disease or a treatment protocol. Together, these factors open the path for an easier and safer evaluation of new radiolabeled substances. Furthermore, delayed images up to 30 days post injection of, e.g., Zr-89-labeled molecules [9] will give completely new insights into the biodistribution of new (radio-)pharmaceuticals. These qualities will open the door for new indications not presently feasible with the current state of the art PET/CT scanners.

An up to 40-fold higher sensitivity [5] can translate to an up to 40 times shorter image acquisition. The current state of the art scanners require 5 to 15 min acquisition time for good image quality, depending on the patient size, the ROI, the age, and technology of the scanner. The new generation of TB PET scanners can generate the same quality of data in only 30 s while maintaining the image quality and showing now significant differences regarding the identification of target lesions [1]. Potential real-life scenarios in which this significantly reduced acquisition time can be of clinical relevance include (1) breath-hold acquisition (no motion-correction needed), (2) avoidance of anesthesia in children or demented patients, (3) higher comfort for patients with pain, and (4) obviously a high patient throughput in high-volume centers. The latter however seems of less relevance, as the patient handling time, i.e., putting the patient on and off the scanner, is already the major contributor to the time required per patient in PET scanning.

The fact that the increased sensitivity can be employed to reduce the amount of injected activity and consequently decreasing the corresponding PET-associated radiation dose seems to be of higher relevance from a clinical as well as from a molecular imaging research point of view. Whereas currently around 200 to 370 MBq of the standard tracer fluordeoxyglucose (FDG) is injected, the TB PET might allow a reduction to 20 to 40 MBq FDG and below [14, 15], which results in a significant reduction of the corresponding FDG-related radiation dose (from around 4.0 to 7.4 mSv to 0.4 to 0.8 mSv) [26, 85]. A 10-fold activity reduction resulting in maintained image quality and kinetic information was shown for healthy individuals undergoing examination with the uExplorer [57]. This tremendous reduction of the radiation dose is especially of relevance for not only pediatric patients, but also young adults. Moreover, for benign diseases (e.g., inflammatory, cardiovascular, orthopedic) currently less commonly referred to PET and even for screening, the use of TB PET could become a valuable option. With the prospect of a TB FDG PET including a low-dose CT being available for a radiation dose below 2 mSv, the patient scope can be expanded to include evaluation of benign diseases and screening applications. An additional aspect is the use of PET for the monitoring of disease phase transition as well as the monitoring of therapies. The low radiation exposure would allow for a frequent monitoring in these circumstances.

Whereas faster image acquisition and reduction of radiation dose are straightforward benefits of TB PET, the even more promising clinical relevance is the expansion of PET to new applications. Such new applications, discussed in more detail in the future application section, address the option of dynamic total-body scanning (easy estimation of parametric images based on the very early dynamics following FDG injection) [33], the assessment of multi-organ disease, the opportunity to consider maternal-fetal medicine, but above all the use of PET for drug development including studies of pharmacokinetics (PK) and pharmacodynamics (PD). This technology could be a very valuable tool for the theranostic concepts in personalized medicine approaches and might prove to facilitate significantly the translation of new radiotracers into clinical application.

## Future perspective in total-body PET/CT and total-body PET/MR

Besides the immense increase in sensitivity and the diagnostic value that is associated with the first generation of TB PET/CT systems, there remain some challenges to be faced. However, if overcoming issues regarding patient comfort and space requirements, there are even more potential fields of application imaginable, including a strong involvement of artificial intelligence (AI).

### Remaining challenges

Next to all the exciting opportunities that arise with TB PET systems, patient compliance is to be considered as one of the remaining challenges of TB PET, since claustrophobia will be a more frequently observed issue due to the long bore of the scanner, however comparable to current MRI scanners. Furthermore, it will be important to have a solution for a fast access to the patient in case any emergency arises, e.g., a contrast agent incident. If dynamic scans are acquired, the CT dose for the attenuation correction should be acquired at ultra-low doses to allow frequent repetition. At the same time, motion correction should also be considered and ideally compensated by means of dedicated, AI-supported reconstruction software. Efforts have to be made to develop ultra-fast image reconstruction algorithm that can keep up with the increased speed in which this highly sensitive systems acquire data. Although PET-guided biopsies are certainly not frequently used, TB PET scanners will not allow to perform this within the scanner. Adding a word of caution to the exciting spectrum of TB PET-associated clinical opportunities: The clinical benefit and ideally an additional cost-benefit have to be prospectively shown to justify the higher costs of the TB PET systems compared to current state of the art scanners (an approximately 4 to 5 times higher initial investment). From a feasibility point of view, also the installation of the scanners itself has to be considered. The uExplorer has a rather large footprint and will not fit in every current department, while the recently introduced Biograph Vision Quadra has a footprint similar to a conventional clinical PET/CT scanner (see Fig. 4c). Coming along with a higher detection efficiency and lower costs, this is why the use of BGO as scintillator material could be re-inforced in future PET/CT systems—if appropriate photo-sensors and readout electronics can be employed to improve its timing performance (see also the section “Scintillators”).

### On the perspective of total-body PET/MR systems

**Fig. 6:**
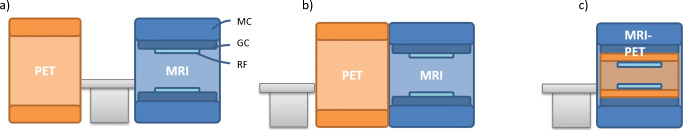
Integration options of TB PET (orange) with an MRI system (blue). **a** TB PET and MRI share the same table, but are two separate systems. This system shows the least interference due to the low B0 stray field at the PET location; **b** TB PET and MRI aligned, with medium interference due to strong B0 stray field; **c** simultaneous acquisition of TB PET and MRI with strong interference of PET with the B0, gradient, and RF fields of MRI

In contrast to the mentioned TB PET/CT systems, to this day, no commercial or research TB PET/MR system has been announced. While for PET/CT systems, increasing the aFOV of a PET system can be achieved by adding detector rings to the PET gantry, scaling an organ-based or WB PET/MRI system to a TB PET/MRI system is a less trivial task, depending on how the TB PET is integrated with the MRI. Figure 6 shows several ways in which TB PET can be combined with MRI. Figure 6a and b show sequential imaging techniques that facilitate system integration as in PET/CT at the expense of a sequential workflow. For simultaneous TB PET/MRI, as indicated in Fig. 6c, innovative concepts of realizing larger homogeneous magnetic fields would have to be developed to match the FOV of PET and MRI on a much larger scale than nowadays possible. This would of course be a major challenge since the FOV of an MRI system is usually a rotational ellipsoid or a sphere with a diameter of 50 to 55 cm. The aFOV would have to be increased to about 1 m at least to meet the condition of a TB MRI. While this would of course be feasible from a physical point of view and interesting from a medical point of view, it is questionable from an economic point of view. For the latter aspect, the recent commercial development of large-bore low-field MRIs ($\sim $ 0.6 T) represents an interesting development, resulting in reduced costs and footprint size [59].

Nevertheless, even with an unchanged MRI FOV, the combination of TB PET with MRI would be of particular interest, because MRI—compared to CT—does not imply any additional dose for the overall examination. On top, the organ-related high sensitivity gain of TB PET would be of advantage here. In addition, highly accurate compensation of motion within the body during TB PET/CT imaging remains a challenging task. This would be an advantage of simultaneous TB PET/MRI because it allows continuous measurements of internal organ motion not captured by PET/CT. Apart from integration aspects such as magnetic field uniformity and coil limitations, matching the MR sequences to the throughput rates and the overall workflow of the highly sensitive TB PET systems and its potentially reduced scan time has to be addressed as well. Since MRI scans usually take 20 to 40 min, new methods need to be developed that allow rapid imaging of volumes many times (3–4) those of current MRIs. Apart from the immense technical effort, the clinical need for a TB PET/MR system has to be investigated. This is especially not uncritical because whole-body PET MR scans have found less application in clinical practice than expected.

However, the ultra-low dose capability of a future TB PET/MRI system could perhaps change this. In contrast, recent approaches to AI-assisted CT reconstruction are expected to enable very low-dose CT acquisitions [61, 99]. Furthermore, as for a larger axial FOV of the PET system costs are dominated by the PET system, TB PET/MRI and TB PET/CT are getting close with respect to their system, installation, and maintenance costs, which is why TB PET/MRI systems might be seen as an evolution of TB PET/CT in the next years to come. This is supported by the ultra-low dose capability of a future TB PET/MRI system, with its outstanding ability for high-accuracy motion compensation. So, it remains open whether this development will occur or not.

### The role of machine learning in total-body PET/CT

The amount and quality of data in a TB PET/CT examination are expected to be higher than those in conventional PET/CT measurements. Efforts to reduce the required storage space by reducing the administered dose have shown that even smaller data files of TB PET scanners show an increased percentage of effective counts to be evaluated [57]. In addition, the data acquisition process itself will be different compared to current WB PET/CT scanners as larger aFOVs will be acquired simultaneously. This enables a wide range of potential applications where ML can be a great support for correction and automation [111]. Since in TB PET/CT the administered radiation dose will often be dominated by the CT component compared to conventional PET/CT, low-dose techniques that allow repetitive studies would be of great advantage. Therefore, a promising domain for ML methods are the areas of low-dose CT or PET-only measurements, where the dose of CT could be largely or even completely avoided by using methods of DL [3, 24, 45, 50]. Promising work has already been published in the field of attenuation and scatter correction. For attenuation correction, cycle-consistent generative adversarial networks (CycleGAN) were used to map the non-attenuation-corrected to the attenuation-corrected PET image [31]. Convolutional neural networks (CNNs) have been proposed for scatter correction of PET data, which is not necessarily limited to TB PET images [10]. Furthermore, there is an enormous need to improve the overall robustness and quantification of quantitative PET by reducing registration and motion artifacts, which will be a major research area in the coming years.

### Future applications

The tremendously increased sensitivity of the TB PET will not only allow for faster imaging and reduced radiation doses but also open up a number of new clinical and research applications. The long existing dream of truly quantitative PET imaging, ultra-fast dynamic imaging, and total-body dynamic imaging, as well as antibody-PET imaging can be realized. Potential applications and required next steps are discussed in this paragraph.

A comparably straightforward application is the use of TB PET for drug development. Currently, an estimate of 14% of new drugs in clinical trials are eventually granted FDA approval [23]. One of the reasons for the high failure rate is a lack of understanding how the investigational products interact within the body. The use of radiolabeled versions of these drugs and the availability of TB PET will allow for a better understanding of the biodistribution and its change over time. Currently, only very few pharmaceutical companies use PET for the evaluation of their drugs’ PK and PD [75]. Especially in oncologic therapies, and also in neurological diseases, many new targeted therapies have been introduced in the last decade [43, 56]. This so-called potential of total-body dynamic imaging can improve the understanding of PK and PD of investigational drugs and reduce the risk in development. Another future application of total-body dynamic imaging is combining this huge amount of imaging data with clinical parameters and employing AI. Including deep learning networks, an AI could be trained based on curated outcome information to predict histopathology, prognosis, or even outcome of oncologic, cardiovascular, or neurological diseases [47, 48]. The high sensitivity of the system opens the path for the clinical introduction of radionuclides with long half-lives, e.g., to evaluate individual PD and PK of antibodies, nanobodies, or affibodies that are used for targeted therapies such as applied in breast cancer [2, 44].

Further areas of interest lie in the field of maternal-fetal medicine, which until today due to comparably high radiation and long image acquisition have not yet been profoundly explored for PET imaging. Furthermore, the modulation of brain oxygen use over time remains an often critical clinical question and could be studied sequentially with TB PET [5, 89]. In addition, the recent clinical interest focusing on the interconnectivity of various organ systems, such as the brain-heart or the brain-gut axis, underlines the potential utility of TB PET to image multiple organ systems in parallel. In this regard, studies on the *β*-amyloid distribution using dynamic WB PET are promising [32].

## Conclusions

The concept of TB PET/CT has shown to be technically feasible. So far, three human TB PET/CT scanners comprising the PennPET Explorer, the uExplorer, and the Biograph Vision Quadra have been realized. The latter two were made commercially available by United Imaging Healthcare, Shanghai, China, and Siemens Medical Solution USA, Knoxville, Tennessee, USA. A human TB PET/MRI has not been developed to this date.

TB PET systems benefit greatly from their increased system sensitivity due to their larger aFOV while remaining the TOF performance of their smaller predecessors. However, it has been shown that this effect saturates for gantry lengths larger than 1 m and is reduced for organ-dedicated compared to total-body imaging [25, 27, 101].

Due to their up to 40-fold increased system sensitivity, TB PET/CT systems result in the opportunity of reforming conventional PET imaging from long scans requiring a high tracer dose and the acquisition of the patient body in several bed positions to short, single-shot, and low-dose scans. This enables more frequent PET/CT scans and possibly screening for preventive examinations, e.g., for cancer or for Alzheimer’s disease. Furthermore, the high sensitivity enables research to investigate the PK and PD of drug delivery down to a new time scale. The enormous amount of acquired data creates the possibility of training deep neural networks for diagnostic purposes. Not only oncology, but also cardiology and neurology are likely to benefit from these developments. From a technical point of view, the increased sensitivity along with maintained TOF performance and SNR could lead to the manifestation of new detection and reconstruction techniques, such as positron annihilation lifetime spectroscopy (PALS), allowing to discriminate between healthy and malignant tissues [71, 122]. For PALS, the exact reconstruction of the point of annihilation is necessary to assign the lifetime of a positronium atom allocated in void tissue space before its decay to the respective tissue. TB PET systems realizing a high spatial resolution and a low SNR are expected to be beneficial for precisely determining the origin of the two annihilation *γ*-photons detected as coincidence. At present, this technique is not applied in PET systems yet.

In summary, the availability of the TB PET technology triggers a number of immediately realizable applications, not only opens up the opportunity for prospective on the obvious lying research questions (both discussed above) but will also open the path for the development of new applications we are currently not yet thinking of. In contrast to the integrated PET/MRI technology, which has not shown the groundbreaking blockbuster indications [115], we think that this new TB PET technology, especially the Biograph Vision Quadra providing a high sensitivity increase while keeping the spatial requirements at a level that is already met at most clinical sites, has much more potential applications that could make it irreplaceable in a lot of clinical applications.

## Abbreviations

aFOV: axial field of view
ADC: analog-to-digital converter
AI: artificial intelligence
APD: avalanche photo-diode
ASIC: application-specific integrated circuit
BGO: bismuth germanate
CBM: continuous-bed-mode
CT: computed tomography
CNN: convolutional neural network
CRT: coincidence resolution time
CycleGAN: cycle-consistent generative adversarial networks
dE/E: energy resolution
DAPS: data acquisition and processing server
DL: deep learning
DOI: depth of interaction
dSiPM: digital silicon photomultiplier
FDG: fluorodeoxyglucose
HR: high resolution
LOR: line of response
LSO: lutetium oxyorthosilicate
LYSO: lutetium yttrium oxyorthosilicate
ML: machine learning
MRI: magnetic resonance imaging
NECR: noise equivalent count rate
NEMA: National Electrical Manufacturers Association
PALS: positron annihilation lifetime spectroscopy
PDPC: Philips Digital Photon Counting
PD: pharmacodynamics
PET: positron emission tomography
PK: pharmacokinetics
PIN: positive-intrinsic-negative
PMT: photo-multiplier tube
QE: quantum efficiency
RF: radiofrequency
ROI: region of interest
SiPM: silicon photomultiplier
SNR: signal-to-noise ratio
SPAD: single-photon avalanche diode
SPECT: single photon emission computed tomography
SS: step-and-shoot
TB: total-body
TDC: time-to-digital converter
tFOB: transaxial field of view
TOF: time-of-flight
WB: whole-body
